# Of artificial intelligence, machine learning, and the human brain. Celebrating Miklos Palkovits' 90th birthday

**DOI:** 10.3389/fnana.2024.1374864

**Published:** 2024-05-03

**Authors:** Denes V. Agoston

**Affiliations:** Department of Anatomy, Physiology and Genetics, Uniformed Services University, Bethesda, MD, United States

**Keywords:** machine learning (ML), artificial neuronal network (ANN), neurotransmitters, energy consumption, efficiency

The promises and challenges of artificial intelligence (AI), machine learning (ML), and deep learning (DL) are based on the premise that we can build machines and write algorithms that will mimic and even surpass the capacity and capabilities of the human brain (Alzubaidi et al., 2021). AI uses artificial neural networks (ANNs) that intend to mimic the works of the neural networks of the human brain. In AI, the strength of the connection of each “neuron” to its “neighbor” is a parameter known as “weight”. The network starts with random “weights” and adjusts them until the output agrees with the correct answer during the “training,” which includes “reading” huge volumes of the text in which some words are masked and then “asking” the network to “guess” what those masked words are. Using over 3 billion words, the network “learns” what the masked words are (Jain et al., 1996). By comparison, an average child requires 3,000 times fewer words to learn and speak a language (Saenko, 2020). It should be noted, however, that a child needs much longer time, ~4 to 5 years to learn ~3,000 words. Regardless of this, as illustrated by generative AI, e.g., ChatGPT and Google's Bard, such “brute force” works well for certain brain functions, i.e., storing and analyzing and finding correlations in massive amounts of existing data (Polyportis and Pahos, 2024).

The current AI comes, however, with caveats. One is the abovementioned inefficiency of ANNs—even a large language model (LLM)—to “learn.” The other one is the currently limited ability of AI for intuition and creativity as compared to the human brain. This is despite the landmark 2016 victory of Google's AlphaGo that beat the South Korean Go champion, Lee Se-dol (Metz, 2016). A third and critical issue is the enormous energy required by generative AI (Saenko, 2020; de Vries, 2023). Training an ANN, i.e., reading through vast amounts of data until the system “understands it,” needs electricity that can be as much as a small country's electricity consumption. Currently, ~2% of the total and global electricity production is used by data centers. In addition, this is only the very beginning of AI. With the predicted growth of AI—assessed by counting the annual rate of increase in chip production, e.g., by NVIDIA—electricity demand for AI will increase dramatically. By some estimates, the global electricity demand for AI and related computing can increase by 85–134 TWh annually. Such an increase in electricity demand is similar to that in Sweden, which doubles its electricity consumption yearly (de Vries, 2023). The effect of such an increase in electricity demand on the “carbon footprint” with the current mix of electric power generation (natural gas: 38%; coal: 22%; nuclear: 19%; renewables: 20%; hydroelectric 6%) can be alarmingly high (Dhar, 2020; Heikkilä, 2023). For example, creating GPT-3 needs 1,287 MWh of electricity with an added 552 tons of CO_2_–or equivalent—and this is before any user has started any queries (Patterson et al., 2021). It is no surprise then that Microsoft has been interested—and has invested—in nuclear power generation, especially in small modular reactors (SMRs) that will not increase the carbon footprint (McFadden, 2023). Microsoft has also invested in Helion—a Sam Altman-backed company—that plans to generate electricity using futuristic, nuclear fusion-based power (Gardner, 2023).

We compare the massive hunger for energy by AI with that of the human brain. While it is hard to calculate the exact energy required by the human brain for its various functions including information processing and analysis, it is clearly only an insignificant fraction of that of AI. In 1989, Ralph Merkle published his study “Energy Limits to the Computational Power of the Human Brain” (Merkle, 1989). He estimated that the human brain uses only ~10 W of energy per second. However, he also estimated that the “computational power” of the human brain is limited to ~10^13^ to 10^16^ operations per second. Regardless of the exact energy “consumption” of the human brain per operation, which is rather challenging to determine even with magnetic resonance spectroscopy (MRS) and functional magnetic resonance spectroscopy (fMRS) (Rothman et al., 2011, 2019; Hyder and Rothman, 2012), the notion that the human brain is using less energy when compared to AI is hard to contest (Hughes, 2023).

A potential cue for such a highly energy-efficient “operation” may be the “wiring”; neuronal connectivity is a critical but not the only aspect of how the human brain operates (Gebicke-Haerter, 2023). In contrast to the computers— and thus AI's—binary *modus operandi*, the human brain is an incredibly complex “machine” using both analog and digital modes simultaneously (Guidolin et al., 2022; Marcoli et al., 2022). The seamless integration and utilization of digital and analog “modes” are likely the “secret” to the unparalleled capacity and abilities of the human brain. Its “operation” is not restricted to binary signaling but to a highly sophisticated and complex combination of electrical and chemical signaling within the networks. The dozens of neurotransmitters and neuromodulators along with their receptors, ion channels, and intracellular “effectors” promote the fact that the human brain is such an incredibly energy-efficient “computer.” In addition, neurons can use more than one neurotransmitter (Svensson et al., 2018), integrating various signaling modalities (e.g., Agoston et al., 1988, 1994). Knowledge of the neurotransmitters and neuromodulators utilized by various human brain regions and neuronal pathways—the chemical neuroanatomy—is fundamental to our understanding of how the human brain operates in health and the chemical changes underlying neuropsychiatric disorders (Hokfelt et al., 1984).

Miklos Palkovits has made an enormous contribution to this field. Miklos, along with Tomas Hokfelt, another significant contributor to the field of chemical neuroanatomy (Hokfelt, 2010) along with other giants of neuroscience—Kjell Fuxe (e.g., Steinbusch, 1981; Rakic, 1988; Sawchenko, 1998; Greengard, 2001; Agnati et al., 2011; Saper and Fuller, 2017; Swanson, 2018) to name a few—have majorly contributed to the “chemical mapping” of the human brain, thus helping us understand its majesty—and mysteries. The Handbook of Chemical Neuroanatomy, first published by editors Bjorklund and Hokfelt in 1983 has reached 22 volumes (Bjorklund and Hokfelt, 1996).

While celebrating Miklos' 80th birthday, 10 years ago, I wrote a short article entitled: “Great insight created by tiny holes; celebrating 40 years of brain micropunch technique” (Agoston, 2014) that summarized his immense contribution to neuroanatomy—up to December 2013. By 2013, Miklos had published more than 1,000 research papers—many of his papers are citation classics, 59 book chapters, and eight books, nominated twice for the Nobel prize. Ten years later, in December 2023, I had the honor of attending Miklos' 90th birthday celebration just to learn about his current and—yes—future projects. During the last 10 years, Miklos has published 57 peer-reviewed papers, numerous book chapters, and reviews and has written and constantly updated his book *Practical Neurology and Neuroanatomy* (co-written with Dr. S. Komoly) with the newest neuroimaging and neurophysiology findings.

Miklos' current research working with collaborators across the globe includes the characterization of the human brain (g)lymphatic system (Mezey and Palkovits, 2015; Mezey et al., 2021), identifying SARS-CoV-2 entry sites into the human brain (Vitale-Cross et al., 2022), identifying the role of neuropeptides and their signaling in neuropsychiatric disorders (Barde et al., 2016, 2024; Hökfelt et al., 2018; Zhong et al., 2022; Samardžija et al., 2023; Vas et al., 2023), and neurogenetics (Roy et al., 2017; Dóra et al., 2022; Hardwick et al., 2022).

The last decade of neuroscience research utilizing powerful imaging, electrophysiology, and such techniques has greatly expanded our knowledge; however, we are still far from a complete understanding of how the human brain works. What are the neurobiological, neuroanatomical, and chemical substrates of consciousness, inspiration, and intuition? What we do know is that Miklos' work has been paving the way toward a better understanding of the marvel, the human brain.

Miklós, thank you for teaching and inspiring so many of us, happy (belated) 90th birthday, and I am so much looking forward to learning much more from you!

## Author contributions

DA: Writing – review & editing, Writing – original draft.
